# Effects of material hardship on depression among adults in South Korea: insights from by the Korea Welfare Panel Study 2008–2017

**DOI:** 10.1186/s12939-021-01531-1

**Published:** 2021-09-07

**Authors:** Soo Hyun Kang, Selin Kim, Eun-Cheol Park, Sung-In Jang

**Affiliations:** 1 Department of Public Health, Graduate School, Yonsei University, Seoul, Republic of Korea; 2 Institute of Health Services Research, Yonsei University, Seoul, Republic of Korea; 3 Department of Preventive Medicine, Yonsei University College of Medicine, 50 Yonsei-ro, Seodaemun-gu, Seoul, 03722 Republic of Korea

**Keywords:** Depression, Socioeconomic status, Material Hardship

## Abstract

**Background:**

Low socioeconomic status deemed by income-based measures is a risk factor for depression. Material hardship is commonly used as a multidimensional socioeconomic indicator to identify the struggles that low-income households encounter that are not captured by conventional income-based measures. The aim of this study was to examine the effect of material hardship on depression.

**Methods:**

We used wave 3 (2008) to wave 12 (2017) panel data collected by the Korea Welfare Panel Study. The material hardship measure included six dimensions: food, housing, medical care, paying utility bills, education, and financial hardship. Depression was measured using the Center for Epidemiologic Studies Depression Scale (CESD-11). A generalised estimating equation model was applied to test the causal association between material hardship and log transferred CESD-11.

**Results:**

The first time point comprised 3,866 participants. Those who continually experienced material hardship had higher depression scores (male: β = 2.82, female: β = 3.98, *p*-value: < .0001). Food hardship was the most critical risk factor (male: β = 3.29, female: β = 4.05, *p*-value: < .0001).

**Conclusions:**

Material hardship is associated with increased risk of depression, especially food hardship. We should consider guaranteeing food security, and community and policy makers should consider material hardship in their approach when identifying low-income populations at high risk for depression.

## Background

South Korea has witnessed a dramatic increase in income inequality and poverty in the past two decades, with the Gini coefficient skyrocketing from 0.28 to 0.35 within this short period of time [1]. As the proportion of females and irregular workers (contractors) in labour market have become higher, their lower income compared to males and regular workers are related to income inequality [2]. This inequality has led to concerns regarding poverty and health inequality in low-income households. Aging society is another cause [2]. Korean government has poverty reducing systems, such as the National Basic Livelihood Guarantee System (NBLGS). However, the proportion of beneficiaries is generally only 3% in population because of the strict criteria regarding income level and absence of supportable family members. The rate of beneficiaries in population whose income level met the criteria was only 52.61% which is assumed as the huge blind spot of the system [3]. Hence, the public social spending of GDP in Korea was only 12.2% in 2019, while the average of Organisation for Economic Co-operation and Development (OECD) was 20.0% [4].

Socioeconomic status, which is typically measured by income, has the advantage of providing an objective measure and definition of low-income households. However, income can overlook the daily struggles that these households may experience and only indirectly estimate what low income actually means for people [5, 6]. As numerous studies have highlighted various limitations of the one-dimensional and conventional simplistic income-based measures of socioeconomic status, material hardship (MH) was introduced to capture the multidimensional aspect of the financial challenges people encounter daily [7].

MH was developed by Sen [8] and Townsend [9, 10] to measure the living conditions of the poor. They argued that different groups of people and households have different consumption habits and face different demands. Beverly [11] defined MH as the failure to be able to consume minimal levels of basic services and goods such as food, housing, basic goods, clothing, and medical care mainly due to financial challenges. Researchers have found that there was little overlap between the groups of people deemed poor by the income-based measure and those deemed poor by the MH measure [12]. Thus, MH is used as a socioeconomic indicator to identify the struggles that low-income households actually encounter, unlike the conventional income-based measure.

The World Health Organization issued a report on mental health which stated that ‘No group is immune to mental disorders, but the risk is higher among the poor, homeless, the unemployed, and persons with low education’ [13]. Heflin and Iceland [14] reported a strong relationship between MH, especially when encountering problems with paying bills and having one’s phone disconnected, and depression in the U.S. data. In Korea, numerous researchers have reported the association between low socioeconomic status and mental disorders, such as depressive symptoms [15, 16] and suicidal ideation [17] and attempts [18]. However, in Korea, one-dimensional and conventional income-based measures of socioeconomic status have been much more commonly used than multidimensional measures in studies examining the association between mental disorders and low socioeconomic status.

Although, previous studies have shown the relationship between poverty and depression, in Korea, their relationship to MH, which is an extended concept of poverty, and depression has not been thoroughly assessed. Therefore, the aim of this study was to examine the effect of MH on depression.

## Methods

### Data and population

We used data from the Korea Welfare Panel Study (KoWePS) conducted by the Korea Institute for Health and Social Affairs and Seoul National University. The participants were recruited using two-stage stratified cluster sampling in 2006. These data are considered suitable for consideration in low-income policies or poverty studies because approximately 50% of the sample were low-income earners with a median income of 60% or less. The present sample was drawn from waves 3 (2008) to 12 (2017) of the study. The 2009 baseline data included 16,255 individuals from 6,207 households.

To analyse the effect of MH, we excluded participants under 19 years of age (3,679 excluded) and individuals with missing data (8,130 excluded). In addition, 580 individuals who did not respond to the MH questions in the prior year (2008) were excluded. Thus, the final 2009 data included a total of 3,866 individuals: 3,568 males and 298 females.

### Measures

The primary outcome was depression, which was measured based on the 11-item version of the Center for Epidemiologic Studies Depression Scale (CESD-11). The CESD-11 is a shorter version of the original 20-item version and is a self-reported screening tool which is well validated [19]. In KoWePS, respondents reported symptoms they had experienced during the previous week using a version of the scale that had been translated into Korean and validated by Cho and Kim [20]. The scale used a four-point Likert scale that ranged from 0 to 3, and the total score was calculated by adding up all responses. Higher scores indicated greater distress; a score above 16 is usually considered indicative of depression [21].

The MH was the key independent variable. In KoWePS, the participants answered 13 questions on whether their household was affected by MH in the prior year. The questions covered the following areas: (1) ran out of food and could not afford to buy more, (2) could not afford balanced meals, (3) adults in the household skipped meals or did not have enough to eat, (4) ate less than needed because could not afford to buy enough food, (5) were hungry but did not eat because could not afford to buy food to eat, (6) had electricity, telephone, or water disconnected because of unpaid bills, (7) unable to pay utility bills before the due date, (8) unable to pay rent for over two months or had to move out because of unpaid rent or inability to pay rent, (9) unable to sufficiently heat the home during the winter, (10) myself or other family members needed to see a doctor but could not afford to go, (11) unable to pay the national health insurance premium and lost eligibility for national health insurance, (12) had problems with credit, and (13) unable to pay children’s public education tuition. All items were binary variables (Yes = 1; No = 2) except two of the food-related hardship items (questions 1 and 2), which had three responses (i.e. ‘often’, ‘sometimes’, and ‘never’). We coded them as binary indicators by combining the ‘sometimes’ and ‘often’ responses into one category. The 13 MH items capture the four dimensions of MH (i.e. food hardship, difficulty paying bills, lack of medical care, and unstable housing) [7, 14], which have been widely used in research. The unique aspects of MH in the South Korean context, such as having problems with credit and the inability to pay children’s tuition, were also included.

If participants answered ‘Yes’ to at least one of the 13 questions, we considered they had experienced MH. A change in MH over one year was classified into four categories: (1) ‘No → No’ for not having experienced MH for the whole study period; (2) ‘No → Yes’ for not having experienced MH in the prior year but having experienced MH in the following year; (3) ‘Yes → No’ for having experienced MH in the prior year and not having experienced MH in the following year; (4) ‘Yes → Yes’ for having experienced MH in both the prior year and following years of the study.

Demographic, socioeconomic, and health related factors were included. Demographic variables included gender, age, and region. Socioeconomic variables included education level, marital status, NBLGS beneficiary status, and income level (quartile). Health related factors included smoking, alcohol intake, disability status, prior year depression status, and chronic diseases.

### Statistical analysis

We calculated the distribution of the participants’ general characteristics at baseline. Analysis of variance (ANOVA) was used to assess the differences in mean CESD-11 scores among the 3,866 sampled subjects at the first time point (2008 → 2009). To analyse the effect of MH on depression using the repeated measured panel data, we used the generalised estimating equation (GEE) model with exchangeable correlation matrix. The GEE model is more efficient and provides unbiased regression estimates for use in analysing repeated measures research designs with non-normal response variables [22]. The GEE model considers the correlation within the subjects to yield the regression coefficient (β), the standard error of the coefficient (SE), and the corresponding *p*-value by doing We evaluated whether the MH transition in each prior year was associated with their depression in each subsequent year over two consecutive years from 2008 and 2009 till 2016 and 2017. Thus, total nine time points were used in this study. Statistical analyses were performed using the GENMOD procedure with ‘repeated subject’ option in SAS, version 9.4 (SAS Institute, Inc., Cary, NC, USA). The results were considered statically significant if the *p*-value was less than 0.05.

## Results

Table 1 presents participants’ general characteristics at the first change time point (2008 → 2009). Of the 3,866 participants, 71.2% of the male and 47.7% of the female participants were in the No → No group. The No → Yes group was the smallest, accounting for 6.8% of the male and 6.7% of the female participants. Male participants in the Yes → Yes group showed the highest mean score on the CESD-11 (No → No: 6.79; No → Yes: 10.86; Yes → No: 9.56; Yes → Yes: 12.77). Female participants in the No → Yes group showed the highest mean scores on the CESD-11 (No → No: 14.14; No → Yes: 19.18; Yes → No: 15.27; Yes → Yes: 17.95). Generally, female participants demonstrated higher mean scores on the CESD-11 than males.

**Table 1:** Participants' general characteristics at the first change time point (2008 → 2009)

**Variables**	**CESD-11**											
	**Male**					***P* value**	**Female**					***P* value**
	**N**	**%**	**MEANS**	±	**SD**		**N**	**%**	**MEANS**	±	**SD**	
**TOTAL**	**3,568**	**100**	**8.02**	±	**9.06**		**298**	**100**	**15.77**	±	**12.57**	
**Change in material hardship (2008 → 2009)**												
No → No	2,539	71.2	6.79	±	8.05	< .0001	142	47.7	14.14	±	12.29	0.3803
No → Yes	243	6.8	10.86	±	10.32		20	6.7	19.18	±	14.29	
Yes → No	403	11.3	9.56	±	9.96		50	16.8	15.27	±	12.12	
Yes → Yes	383	10.7	12.77	±	11.08		86	28.9	17.95	±	12.63	
**Prior year depression status (2008)**												
No	2,924	82.0	6.31	±	7.34	< .0001	164	55.0	10.51	±	10.47	< .0001
Yes	644	18.0	15.79	±	11.67		134	45.0	22.20	±	11.96	
**Age (years)**												
19–29	280	7.8	7.03	±	8.88	0.1147	22	7.4	17.44	±	14.97	0.0156
30–39	740	20.7	6.05	±	7.18		21	7.0	9.61	±	10.65	
40–49	752	21.1	7.55	±	9.02		34	11.4	12.78	±	10.82	
50–59	535	15.0	8.44	±	9.74		40	13.4	14.18	±	12.82	
60–69	624	17.5	8.73	±	9.51		48	16.1	21.63	±	13.38	
≥ 70	637	17.9	10.26	±	9.49		133	44.6	15.58	±	11.80	
**Region**												
Metropolitan	1,508	42.3	8.14	±	9.35	0.0617	126	42.3	16.46	±	13.63	0.2671
Rural	2,060	57.7	7.93	±	8.84		172	57.7	15.25	±	11.75	
**Education level**												
Middle school or under	1,299	36.4	9.99	±	9.85	0.1855	225	75.5	16.47	±	12.34	0.0614
High school	1,280	35.9	7.63	±	8.79		44	14.8	14.79	±	13.42	
College or above	989	27.7	5.93	±	7.68		29	9.7	11.79	±	12.67	
**Marital status**												
Living w spouse	2,726	76.4	7.42	±	8.36	< .0001	73	24.5	13.60	±	12.55	0.4768
Living w/o spouse	842	23.6	9.96	±	10.78		225	75.5	16.47	±	12.53	
**Income level**												
Low	840	23.5	10.95	±	10.25	0.0195	111	37.2	18.85	±	12.72	0.1082
Lower middle	867	24.3	8.65	±	9.42		85	28.5	16.49	±	12.15	
Upper middle	883	24.7	7.17	±	8.17		63	21.1	12.78	±	11.57	
High	978	27.4	5.71	±	7.51		39	13.1	10.21	±	12.13	
NBLGS* status												
Non-beneficiary	3,255	91.2	7.38	±	8.39	0.0003	217	72.8	14.38	±	12.06	0.0976
Beneficiary	313	8.8	14.73	±	12.42		81	27.2	19.48	±	13.22	
**Current smoking status**												
Non-smoker	2,159	60.5	8.32	±	9.18	< .0001	195	65.4	15.79	±	12.65	0.4713
Current smoker	1,409	39.5	7.57	±	8.84		103	34.6	15.73	±	12.49	
**Alcohol consumption**												
Non-drinkers	860	24.1	10.35	±	10.34	0.0013	167	56.0	17.00	±	12.08	0.1284
Once a week or less	1,260	35.3	6.97	±	8.34		93	31.2	11.98	±	11.77	
2–3 times a week	825	23.1	7.08	±	8.46		21	7.0	20.00	±	13.80	
More than 4 times a week	623	17.5	8.19	±	8.73		17	5.7	19.14	±	15.85	
**Disability status**												
Not disabled	3,117	87.4	7.45	±	8.59	0.0001	269	90.3	15.57	±	12.65	0.1338
Disabled (no grade, grade 4–7)	161	4.5	14.41	±	11.87		8	2.7	11.82	±	9.72	
Disabled (grade 1–3)	290	8.1	10.56	±	10.31		21	7.0	19.74	±	12.13	
**Chronic diseases**												
No	1,868	52.4	6.49	±	7.61	< .0001	86	28.9	11.63	±	11.93	0.0932
Yes	1,700	47.6	9.70	±	10.16		212	71.1	17.44	±	12.47	

*NBLGS the National Basic Livelihood Guarantee System

Table 2 shows the results of the GEE model analysis of factors associated with depression. The MH Yes → Yes group had higher depression scores (male: β = 2.82, female: β = 3.98) than the No → No group. Moreover, the MH Yes → No group had the higher depression scores as the second (male: β = 2.28, female: β = 3.41), while the MH No → Yes group had only slightly higher depression scores (male: β = 0.45, female: β = 0.84) than the No → No group. Depression scores were higher among participants who experienced depression (having a CESD-11 score of minimum 16) in the prior year (male: β = 3.14, female: β = 2.93) than among those who did not experience depression in the prior year. Participants with a lower income level tended to have higher depression scores (low: male, β = 1.98, female, β = 2.05; lower middle: male, β = 1.00, female, β = 1.13; upper middle: male, β = 0.56, female, β = 0.53) than those with a higher income level. Depression scores were higher among the disabled participants (male: β = 0.96, female: β = 1.09), especially the severely disabled (male: β = 2.62, female: β = 2.05), compared to the non-disabled participants. Further, unmarried participants, NBLGS beneficiaries, current smokers, and participates with chronic diseases tended to have higher CESD-11 scores. Only male participants who consumed alcohol less than 4 times a week (once a week or less: β = -0.59; 2–3 times a week: β = -0.70) had lower CESD-11 scores than non-drinkers.

**Table 2:** Generalized estimating equation (GEE) analysis of factors associated with CESD-11

**Variables**	**CESD-11**					
	**Male**			**Female**		
	**ß**	**S.E**	***P* value**	**ß**	**S.E**	***P* value**
**Change in material hardship**						
No → No	Ref			Ref		
No → Yes	2.28	0.20	< .0001	3.41	0.26	< .0001
Yes → No	0.45	0.16	0.0045	0.84	0.21	< .0001
Yes → Yes	2.82	0.24	< .0001	3.98	0.30	< .0001
**Prior year depression status**						
No	Ref			Ref		
Yes	3.14	0.19	< .0001	2.93	0.18	< .0001
**Age (years)**						
19–29	Ref			Ref		
30–39	0.84	0.19	< .0001	0.19	0.22	0.3794
40–49	1.30	0.20	< .0001	0.02	0.24	0.9372
50–59	1.95	0.24	< .0001	0.76	0.28	0.0068
60–69	2.30	0.26	< .0001	1.09	0.32	0.0007
≥ 70	3.52	0.27	< .0001	2.90	0.33	< .0001
**Region**						
Metropolitan	Ref			Ref		
Rural	-0.17	0.11	0.1190	-0.04	0.13	0.7340
**Education level**						
Middle school or under	0.72	0.19	0.0002	1.44	0.25	< .0001
High school	0.10	0.13	0.4331	0.33	0.17	0.0496
College or above	Ref			Ref		
**Marital status**						
Living w spouse	Ref			Ref		
Living w/o spouse	2.15	0.15	< .0001	1.36	0.16	< .0001
**Income level**						
Low	1.98	0.15	< .0001	2.05	0.18	< .0001
Lower middle	1.00	0.11	< .0001	1.13	0.14	< .0001
Upper middle	0.56	0.09	< .0001	0.53	0.12	< .0001
High	Ref			Ref		
NBLGS* status						
Non-beneficiary	Ref			Ref		
Beneficiary	2.05	0.30	< .0001	1.93	0.30	< .0001
**Current smoking status**						
Non-smoker	Ref			Ref		
Current smoker	0.69	0.10	< .0001	1.72	0.40	< .0001
**Alcohol consumption**						
Non-drinkers	Ref			Ref		
Once a week or less	-0.59	0.11	< .0001	-0.11	0.12	0.3700
2–3 times a week	-0.70	0.13	< .0001	0.09	0.23	0.6883
More than 4 times a week	-0.26	0.16	0.0920	0.59	0.43	0.1741
**Disability status**						
Not disabled	Ref			Ref		
Disabled (no grade, grade 4–7)	0.96	0.22	< .0001	1.09	0.33	0.0011
Disabled (grade 1–3)	2.62	0.40	< .0001	2.05	0.55	0.0002
**Chronic diseases**						
No	Ref			Ref		
Yes	1.02	0.09	< .0001	1.23	0.13	< .0001
**Year**						
2009	Ref			Ref		
2010	-1.10	0.17	< .0001	0.95	0.84	0.2608
2011	-1.52	0.16	< .0001	-0.01	0.82	0.9913
2012	-2.32	0.16	< .0001	-2.45	0.73	0.0008
2013	-1.97	0.16	< .0001	-2.57	0.69	0.0002
2014	-1.09	0.16	< .0001	-1.90	0.69	0.0060
2015	-2.43	0.16	< .0001	-2.96	0.70	< .0001
2016	-2.34	0.17	< .0001	-2.99	0.69	< .0001
2017	-2.28	0.17	< .0001	-3.17	0.69	< .0001

*NBLGS The National Basic Livelihood Guarantee System

Table 3 outlines the subgroup analysis regarding the effects of MH on depression stratified by socioeconomic and health-related variables. Generally, the MH Yes → Yes group had the highest depression status risk, followed by the No → Yes group. Although some were not statistically significant, the Yes → No groups also had a slightly higher risk of depression than the No → No groups. Among male participants who were depressed in the prior year and had a low income level, both the No → Yes and the Yes → Yes groups had higher risk of depression status even compared to the result from Table 2. A*mong female participants who had a low income level, were disabled, had a low educational level, and were suffering from chronic diseases, higher risk of depression status in the No → Yes and the Yes → Yes groups were similarly observed.

**Table 3:** Subgroup analysis of CESD-11 stratified by covariates

**Variables**	**CESD-11**									
	**No → No**	**No → Yes**			**Yes → Yes**			**Yes → Yes**		
	ß*	ß*	**S.E**	***P* value**	ß*	**S.E**	***P* value**	ß*	**S.E**	***P* value**
**Male**										
**Prior year depression status**										
No	Ref	2.13	0.21	< .0001	0.49	0.16	0.0020	2.51	0.25	< .0001
Yes	Ref	2.83	0.66	< .0001	-0.03	0.52	0.9490	3.26	0.56	< .0001
**Income level**										
Low	Ref	3.14	0.36	< .0001	0.62	0.33	0.0621	3.43	0.40	< .0001
Lower middle	Ref	2.44	0.39	< .0001	0.33	0.30	0.2609	2.83	0.40	< .0001
Upper middle	Ref	1.57	0.39	< .0001	0.35	0.30	0.2486	2.06	0.42	< .0001
High	Ref	0.77	0.43	0.0751	0.67	0.33	0.0426	2.56	0.62	< .0001
NBLGS** status										
Non-beneficiary	Ref	2.26	0.22	< .0001	0.54	0.16	0.0010	2.61	0.25	< .0001
Beneficiary	Ref	2.38	0.62	0.0001	0.16	0.56	0.7744	3.06	0.59	< .0001
**Disability status**										
Not disabled	Ref	2.18	0.22	< .0001	0.36	0.17	0.0348	2.80	0.25	< .0001
Disabled (no grade, grade 4–7)	Ref	2.68	0.74	0.0003	0.79	0.55	0.1495	2.66	0.77	0.0005
Disabled (grade 1–3)	Ref	2.40	0.85	0.0050	0.76	0.77	0.3288	3.25	0.96	0.0007
**Chronic diseases**										
No	Ref	1.65	0.26	< .0001	0.42	0.20	0.0387	2.38	0.29	< .0001
Yes	Ref	2.80	0.30	< .0001	0.48	0.25	0.0563	3.22	0.35	< .0001
**Female**										
**Prior year depression status**										
No	Ref	2.74	0.27	< .0001	1.10	0.23	< .0001	2.97	0.32	< .0001
Yes	Ref	5.33	0.59	< .0001	0.34	0.47	0.4687	5.18	0.56	< .0001
**Income level**										
Low	Ref	4.39	0.40	< .0001	0.93	0.35	0.0081	4.92	0.44	< .0001
Lower middle	Ref	3.08	0.49	< .0001	0.74	0.40	0.0662	3.60	0.53	< .0001
Upper middle	Ref	2.25	0.57	< .0001	1.45	0.49	0.0028	1.79	0.65	0.0061
High	Ref	1.89	0.67	0.0045	0.51	0.46	0.2654	4.01	0.92	< .0001
NBLGS** status										
Non-beneficiary	Ref	3.28	0.28	< .0001	0.86	0.23	0.0001	4.17	0.36	< .0001
Beneficiary	Ref	3.62	0.60	< .0001	0.83	0.53	0.1169	3.54	0.56	< .0001
**Disability status**										
Not disabled	Ref	3.38	0.27	< .0001	0.79	0.22	0.0003	3.96	0.31	< .0001
Disabled (no grade, grade 4–7)	Ref	4.15	1.00	< .0001	0.97	0.88	0.2698	4.78	1.26	0.0001
Disabled (grade 1–3)	Ref	3.27	1.27	0.0097	2.40	1.13	0.0338	3.79	1.32	0.0041
**Chronic diseases**										
No	Ref	2.00	0.38	< .0001	0.48	0.30	0.1078	2.37	0.43	< .0001
Yes	Ref	4.17	0.33	< .0001	1.09	0.28	< .0001	4.89	0.38	< .0001

*All other covariates were adjusted

**NBLGS The National Basic Livelihood Guarantee System

Table 4 shows the results of effects of MH on depression based on the dimensions of MH. In the MH sub-categories associated with food, paying utility bills, and financial hardship, male participants who continually experienced MH showed the highest depression scores (food hardship: β = 3.29; paying utility bills hardship: β = 1.53; financial hardship: β = 0.87), while the No → Yes group showed the second highest scores (food hardship: β = 2.46; paying utility bills hardship: β = 1.02; financial hardship: β = 0.68). For housing hardship, the No → Yes group showed higher depression scores (β = 2.02) than the Yes → Yes group. In terms of food, and medical care hardship, female participants who continually experienced MH showed the highest depression scores (food hardship: β = 4.05; medical care hardship: β = 3.63), followed by the No → Yes group (food hardship: β = 3.70; medical care hardship: β = 2.66). As for housing and financial hardship, the No → Yes group showed a higher depression score (housing hardship: β = 3.36, financial hardship: β = 1.09) than the Yes → Yes group.

**Table 4:** GEE analysis of each material hardship dimension

**Material hardship dimensions**	**CESD-11**									
	**No → No**	**No → Yes**			**Yes → No**			**Yes → Yes**		
	ß*	ß*	**S.E**	***P* value**	ß*	**S.E**	***P* value**	ß*	**S.E**	***P* value**
**Male**										
Food hardship	Ref	2.46	0.26	< .0001	0.67	0.21	0.0016	3.29	0.43	< .0001
Housing hardship	Ref	2.02	0.57	0.0004	0.13	0.45	0.7678	1.54	0.75	0.0408
Paying utilization bills hardship	Ref	1.02	0.36	0.0047	0.37	0.25	0.1380	1.53	0.58	0.0083
Financial hardship	Ref	0.68	0.35	0.0478	-0.10	0.28	0.7258	0.87	0.32	0.0065
Education hardship	Ref	0.01	0.72	0.9859	1.35	0.78	0.0817	0.52	1.61	0.7468
Medical care hardship	Ref	1.54	0.48	0.0012	0.28	0.38	0.4718	-0.44	0.84	0.6005
**Female**										
Food hardship	Ref	3.70	0.31	< .0001	1.08	0.27	< .0001	4.05	0.44	< .0001
Housing hardship	Ref	3.36	0.68	< .0001	0.42	0.60	0.4873	3.30	1.15	0.0040
Paying utilization bills hardship	Ref	0.90	0.47	0.0570	-0.19	0.39	0.6189	1.10	0.76	0.1510
Financial hardship	Ref	1.09	0.51	0.0319	0.25	0.36	0.4831	0.90	0.45	0.0430
Education hardship	Ref	0.49	1.08	0.6481	-1.15	0.96	0.2311	-1.58	1.87	0.3978
Medical care hardship	Ref	2.66	0.64	< .0001	0.12	0.52	0.8239	3.63	1.46	0.0133

^*^All covariates were adjusted

## Discussion

In this study, we examined the effect of MH on depression. The main finding of the present study suggests that changes in experiencing MH and constantly experiencing MH are related to an increased risk of depression. A change from Yes to No was found to be slightly related to an increased risk of depression. Both a change from No to Yes and a change from Yes to No were strongly related to an increased risk of depression. Moreover, the MH No → Yes and Yes → Yes groups who were NBLGS beneficiaries, disabled, experienced depression in the prior year, had lower income levels, and chronic diseases were at greater risk of depression compared with those who did not belong to any of these demographic groups, regardless of their gender. Furthermore, food hardship, one of the dimensions of MH, was found to be strongly associated with depression, more than any other factor, while education hardship was not statistically associated with depression. Females were found to be more at risk of depression than males, with females showing higher depression scores than males at the first time point.

In line with this finding, previous studies have shown that MH is an important risk factor for mental health in low-income households [14, 23]. Ross and Huber [24] pointed out that daily struggles to meet basic needs due to limited financial resources may result in experiencing exhaustion, despair, and depression. The previous study used the first two years (2006 and 2007) of KOWEPS to identify the effect of experiencing MH in 2006 on depression status in 2007 using lagged dependent variable model [25]. The result is consistent with our findings showing that MH, especially food hardship, is associated with depression. However, our study identified the transition of experiencing MH over two consecutive years repeatedly using a panel data after adjusting demographic, socioeconomic, and health related factors.

Food hardship had the most salient influence on depression in our study. This subgroup analysis result was similar to the results demonstrated by previous studies [26, 27]. The lack of significant association between depression scores and education hardship and problems with paying utility bills could be explained by the following. First, the sample size for those dimensions may have been too small to determine the nature of the relationship between these factors and depressive symptoms. Second, especially for education hardship, there is generally freedom from financial burdens associated with tuition. In Korea, tuition for public elementary and middle schools are fully supported by the state. Even for higher education, numerous companies subsidise or provide scholarships for their employees’ children as a part of company internal welfare systems.

The effect size of MH on depression differed by gender. In both genders, food hardship was the most influential dimension of MH. This might be related to the gender-role in Korea. Although the participation in the workforce by women has been rapidly growing in Korea, the labor force participation rate of women (52.8%) was distinctly lower than that of men (72.6%) in 2020 [28]. Korean traditions remain strong in families despite many socioeconomic changes. Men regarded domestic work as shameful, defaming masculinity; therefore, doing household chores was treated as a mandatory for women [29]. Because of this “traditional gender-role,” female participants might be much more influenced by MH, especially food hardship, compared to male participants. It might be associated with the different effect size of MH by gender.

One interesting result was that participants who experienced MH in the prior year but no longer experienced MH in the following year still showed higher depression scores than those who had never experienced MH. Stigma and uncertainty about one’s future MH status might be a possible explanation. Mickelson suggested that internalised stigma (individual’s personal negative feelings about their poverty) and experienced stigma (individual’s perception of being treated as stigmatised by behaviours and feelings of others) are related to depression as mediators. Furthermore, internalised stigma and depression were partially mediated by self-esteem and fear of rejection, while experienced stigma was related to depression through fear of rejection only [30]. Our results might reflect the process of suffering those stigmas and recovering from them. The uncertainty about one’s future MH status, which is another possible explanation, may be related to stress. First, pre-experience of social exclusion was found to lead to a blunted cortisol response to stress, which might contribute to higher vulnerability to health disturbances [31]. Second, persistent uncertainty about the future might lead to development of a persistent cerebral energy crisis, contributing to systemic and brain malfunction. When people feel uncertain, they anticipate that outcomes will turn out to be unexpected and feel unable to avoid surprise, and all their cognitive systems strive to reduce it by using cerebral energy. Moreover, the ‘selfish brain’ demands extra energy from the body in times of uncertainty. So, if the brain cannot reduce uncertainty, a persistent cerebral energy crisis may develop into systemic and brain malfunction [32].

The small size of female participants might be related to the self-reported smoking status in this data. In Korea, other self-reported data had inaccurate self-reporting of smoking habits in females [33]. In our study, most female subjects were excluded from the total sample due to the missing value in smoking status. To identify the effect MH change on depression adjusting smoking status in females, further study using data obtained from other smoking detection methods, e.g. urine cotinine examination should be considered.

This study has several limitations. First, because of data limitation, we could not use objective measurement tools for material deprivation such as the Townsend index, which is a widely used and valid measure of material deprivation [34, 35]. However, we used MH measures based on a pervious study [14]. Second, because the KoWePS data largely represent low-income households, the findings are somewhat limited when it comes to generalising to the overall population, particularly high-income households. Third, although we used several control variables to reduce the omitted variable bias, there still might be some unobserved confounding factors that prevent confirming a causal relationship with certainty.

Despite the limitations, some strengths are notable. First, this study was based on the second-largest open access panel survey data in South Korea. Many experts have verified its high validity and national representativeness. As the KoWePS largely represents low-income households and randomly sampled participants nationwide, our findings could be generalised to the South Korean low-income population. Second, to expressly incorporate the financial struggles that low-income households face, we used MH as an index of economic hardship as a substitute for a conventional one-dimensional income-based measure. Most previous studies in Korea have focussed on the association between a type of MH, mainly food insecurity and mental health [36, 37]. However, our study looked at the broader effects of economic difficulties on depression using six dimensions of MH, including two uniquely Korean aspects. Thus, this study contributes to the literature by using MH as a measure of multidimensional financial struggles among low-income households in South Korea.

## Conclusions

In conclusion, this study identified the effects of MH on depression by gender, and the results were significant. We found that people who had experienced MH at least once had a higher risk for depression. Especially, people who continually experienced MH were at the highest risk for depression. Finally, it was found that food hardship was strongly associated with depression. Based on this study, we should consider guaranteeing food security for low-income populations to reduce the incidence of depression. In addition, community and policy makers should consider MH in their approaches to identifying populations at high risk of depression and people encountering financial struggles.

## Data Availability

The dataset is available on the Korea Welfare Panel Study (KoWePS) website: https://www.koweps.re.kr:442/main.do.

## Abbreviations

OECD: Organisation for Economic Cooperation and Development
CESD-11: Center for Epidemiologic Studies Depression Scale
MH: Material hardship
KoWePS: Korea Welfare Panel Study
NBLGS: National Basic Livelihood Guarantee System
ANOVA: Analysis of variance
GEE: Generalised estimating equation
